# Zika might not be acting alone: Using an ecological study approach to investigate potential co-acting risk factors for an unusual pattern of microcephaly in Brazil

**DOI:** 10.1371/journal.pone.0201452

**Published:** 2018-08-15

**Authors:** Monica C. Campos, Jamille G. Dombrowski, Jody Phelan, Claudio R. F. Marinho, Martin Hibberd, Taane G. Clark, Susana Campino

**Affiliations:** 1 Faculty of Infectious and Tropical Diseases, London School of Hygiene & Tropical Medicine, London, United Kingdom; 2 Department of Parasitology, Institute of Biomedical Sciences, University of São Paulo, São Paulo, Brazil; 3 Faculty of Epidemiology and Population Health, London School of Hygiene & Tropical Medicine, London, United Kingdom; CEA, FRANCE

## Abstract

Zika virus infections can cause a range of neurologic disorders including congenital microcephaly. However, while Zika infections have been notified across all regions in Brazil, there has been an unusual number of congenital microcephaly case notifications concentrated in the Northeast of the country. To address this observation, we investigated epidemiological data (2014–2016) on arbovirus co-distribution, environmental and socio-economic factors for each region in Brazil. Data on arbovirus reported cases and microcephaly were collected from several Brazilian Ministry of Health databases for each Federal unit. These were complemented by environmental management, social economic and *Aedes aegypti* infestation index data, extracted from multiple databases. Spatial time “ecological” analysis on the number of arboviruses transmitted by *Aedes* mosquitoes in Brazil show that the distribution of dengue and Zika was widespread in the whole country, with higher incidence in the West-Central region. However, reported chikungunya cases were higher in the Northeast, the region also with the highest number of microcephaly cases registered. Social economic factors (human development index and poverty index) and environmental management (water supply/storage and solid waste management) pointed the Northeast as the less wealthy region. The Northeast is also the region with the highest risk of *Aedes aegypti* house infestation due to the man-made larval habitats. In summary, the results of our ecological analysis support the hypothesis that the unusual distribution of microcephaly might not be due to Zika infection alone and could be accentuated by poverty and previous or co-infection with other pathogens. Our study reinforces the link between poverty and the risk of disease and the need to understand the effect on pathogenesis of sequential exposure to arboviruses and co-viral infections. Comprehensive large-scale cohort studies are required to corroborate our findings. We recommend that the list of infectious diseases screened, particularly during pregnancy, be regularly updated to include and effectively differentiate all viruses from ongoing outbreaks.

## Introduction

Zika, dengue and chikungunya are arboviral diseases transmitted by mosquitoes from the genus *Aedes*. The co-circulation of these viruses in Brazil, sharing the same vectors and causing similar symptoms, represents a major public health challenge. Dengue fever is the most important re-emerging mosquito-borne viral disease worldwide. In Brazil, the incidence of dengue has been frequently high, with 1.49 million cases reported in 2016 [1]. Chikungunya viral infections were first reported in Bahia, Brazil in 2014, in a patient travelling from Angola [2]. Since then, more than 250,000 cases of chikungunya infection have been registered [3]. The first Zika cases reported in Brazil were identified in the Northeast region, in early 2015, in patients presenting symptoms of mild fever, rash, conjunctivitis and arthralgia [4]. Since the first reported autochthonous transmission, more than 200,000 cases have been registered in Brazil [5], mainly during 2015–2016.

The outbreak of Zika infection in Brazil has exposed the high risk this arbovirus imposes during pregnancy to the fetus. Brazil experienced an approximately 20-fold increase in the total number of congenital microcephaly cases from 2014 to 2015, following the confirmation of autochthonous Zika virus transmission [6]. In December 2015, the Brazilian Ministry of Health enhanced congenital microcephaly surveillance by implementing a more sensitive case definition, and added Zika to the list of “TORCHS” pathogens (Toxoplasmosis, Rubella, Cytomegalovirus, Herpes simplex virus, Syphilis) that are screened [7,8]. In February 2016, WHO declared the link between Zika virus and microcephaly to be a Public Health Emergency of International concern. Subsequently, several cases of microcephaly associated with mothers infected with Zika during pregnancy were reported in different Federal Units of Brazil, most of them in the Northeast region [9]. Several studies have shown that the arbovirus can be transmitted vertically during pregnancy and cause congenital problems of the fetus. A recent case-control study performed in Recife, Pernambuco State, revealed that babies with microcephaly were 55 times more likely to have been infected with Zika virus during pregnancy than non-infected [10]. The virus has been detected in the placenta, amniotic fluid and neural tissues of newborns with microcephaly [11–13]. In addition to microcephaly, other birth defects have been reported such as intracranial calcifications and abnormalities of the corpus callosum and the cerebellum, mainly when the exposure to the virus occurs during the first trimester of pregnancy [14].

Before the Brazilian outbreak, no microcephaly-Zika related cases have previously been reported. Zika virus was firstly isolated from a Rhesus monkey in the Zika Forest of Uganda, in 1947 [15]. The first large outbreak of the Zika virus in humans was registered only in 2007 on the Pacific island of Yap, in Micronesia [16]. The same lineage, an Asian type, caused epidemics in the Pacific Islands in 2013–2014 [17–18]. Phylogenetic analyses revealed the Brazilian Zika virus to be closer to those circulating in French Polynesia in 2013, therefore, it might be possible that the Asian-type strains caused unreported microcephaly in the past outbreaks [4,19]. Indeed, a recent retrospective study in French Polynesia has shown that Zika virus infection during the first trimester of pregnancy led to a 1% increase in the risk of congenital microcephaly [20].

Recently, Colombia faced the world’s second largest Zika outbreak, leading to a four-fold increase in the overall microcephaly cases compared to the previous year. However, the relative increase of reported cases (per 10,000 live births) was far fewer than in Brazil, where a nine-fold increase was reported [11]. This observation raises the possibility that additional risk factors might be driving the highest incidence of microcephaly-zika related cases reported so far. It is unclear why a focused cluster of microcephaly cases has occurred in the Northeast region of Brazil. Several theories on the intensification of Zika virus transmission and resulting severe fetal neural defects have been raised. These have pointed to socio-economic factors such as precarious living conditions and low regional gross domestic product (GDP) [21–25]. Further, sequential exposure to arboviruses and even co-infections could play a role in severe clinical manifestations [19, 26–27].

Here we aim to provide insights into the unusual pattern of microcephaly distribution in Brazil. By analysing the number of cases reported and co-distribution of dengue, chikungunya and Zika virus, their vectors, as well as socioeconomic and environmental data, we sought to investigate whether co-acting risk factors might be contributing to the Zika microcephaly cases in the Brazil Northeast region.

## Material and methods

### Mapping the incidence of dengue, chikungunya and Zika virus

To better understand the co-distribution of dengue, chikungunya and Zika virus in Brazil we collated data on reported cases (per 100,000 inhabitants) for all three pathogens from 2014 to 2016. Data including the total number of cases per year and per state for each virus were obtained from epidemiological Bulletins from the Brazilian Ministry of Health database, (until Volume 48, representing cumulative data until week 52 of 2016) [28], confirmed by information available at each Brazil Federal Unit’s Secretary of Health. Bulletins from the Brazilian Ministry of Health database are technical-scientific publications edited by the Department of Health Surveillance, are circulated with monthly and weekly frequency, and are used for reporting the monitoring activities and investigation of specific seasonal diseases. Specifically, they report the total number of cases notified by each State Secretary. The data obtained for Zika in 2015 consist of suspected and/or confirmed cases, as most of them were only confirmed in 2016. Confirmation is based on RT-PCR and serology methodologies. PubMed and Web of Science databases were searched for studies that reported outbreaks of dengue (family Flaviviridae), Zika and chikungunya (family *Togaviridae*) from 2010, including cases of co-infections. The search terms used were: “*Outbreak”*, *“Zika”*, *“Chikungunya”*,*”Dengue”*, *“Brazil”*, and *“Co-infections”*. Maps of arbovirus incidence (per 100,000) per Brazilian Federal Unit were constructed using the package *tmap* in R software [29].

### Distribution of the notified cases of microcephaly

Microcephaly is a congenital malformation where babies are born with a skull size smaller than expected when compared to those of the same sex and age [30]. Specifically, microcephaly is defined as a head circumference that is two standard deviations (SDs) below the mean for the appropriate age and sex, or gestational age if measured at birth. The geographic regions where the microcephaly cases were registered refer to the mother’s place of residence. Data on microcephaly from 2010–2016 were extracted from the Brazilian Ministry of Health website (http://portalsaude.saude.gov.br/) and from the epidemiological Bulletins (until volume 47, week 52 of 2016) available at the Brazilian Ministry of Health database [31]. Additional information was extracted from the System of Strategic Management Support (SAGE) [32], the “*Registro de Eventos em Saúde Pública*” (RESP-Microcefalia) [33]. It includes the epidemiological information (2015–2016) regarding microcephaly and/or Central Nervous System changes, provided under the "*Protocol on Surveillance and Response to Occurrence of Microcephaly and/or Central Nervous System*” [34].

Data on the cumulative incidence of microcephaly from 2015 to 2016 are based on cases that fulfilled the previous definition of cephalic perimeter (33 cm), in addition to the new definitions adopted in the Surveillance Protocol 2015, that defined the 32 cm for boys and 31.5 cm for girls, born with 37 or more weeks of gestation. Notified cases in fetuses, abortions, stillbirths or newborns were confirmed positive for microcephaly by Zika or other undetermined infectious diseases when: a) typical alterations indicative of congenital infection were found, such as cerebral calcifications, ventricular and posterior fossa alterations among other signs observed by an imaging method, or/and b) when confirmed by laboratory-based Zika virus identification. Data are presented per state.

### Socioeconomic data, environmental management

Demographic, socioeconomic, and environmental management data were obtained from the national census performed by the Brazilian Institute of Geography and Statistics (IBGE) [35]. Data for 2016 have been estimated from the last national census (2010). The demographic and socioeconomic variables used, include: (i) *Human development index* (scale from 0 to 1, where lower values indicate lower development), which considers education (average years of studies), longevity (population life expectancy) and gross domestic product per capita; (ii) *Poverty index* (scale from 0 to 100, where lower values indicate greater poverty), which considers health (nutrition and child mortality), education (school attendance) and living standards (sanitation, water, electricity, cooking fuel, assets, house conditions). Environmental management data considers: *Garbage accumulation index* (number of houses with accumulated and uncollected garbage) and *rainwater storage* (number of houses with storage of rainwater in containers). Data are presented per year and per state.

### *Aedes aegypti* surveillance

*Aedes aegypti* infestation levels in Brazil in 2016, was obtained from the *Aedes aegypti* Infestation Index Rapid Survey (LIRAa), performed every year by the Federal Government. This house infestation index measures the percentage of searched buildings with the presence of larvae of *A*. *aegypti*. The average house infestation index (HII) of each municipality is calculated and classified in different risk levels (low-risk or satisfactory, HII < 0.9; mid-risk or alert, 1 < HII < 3.9; high risk of *A*. *aegypti* infestation, HII> 4) [36].

### Statistical analysis

All the data were merged within the R statistical software. We performed an ecological analysis where pairwise relationships between microcephaly, virus infection, social economic factors, arbovirus and *Aedes aegytpi* infestation index were assessed using Spearman’s and Pearson’s correlations and regression models.

## Results

### Distribution of microcephaly and arbovirus infections in Brazil

**Fig 1:**
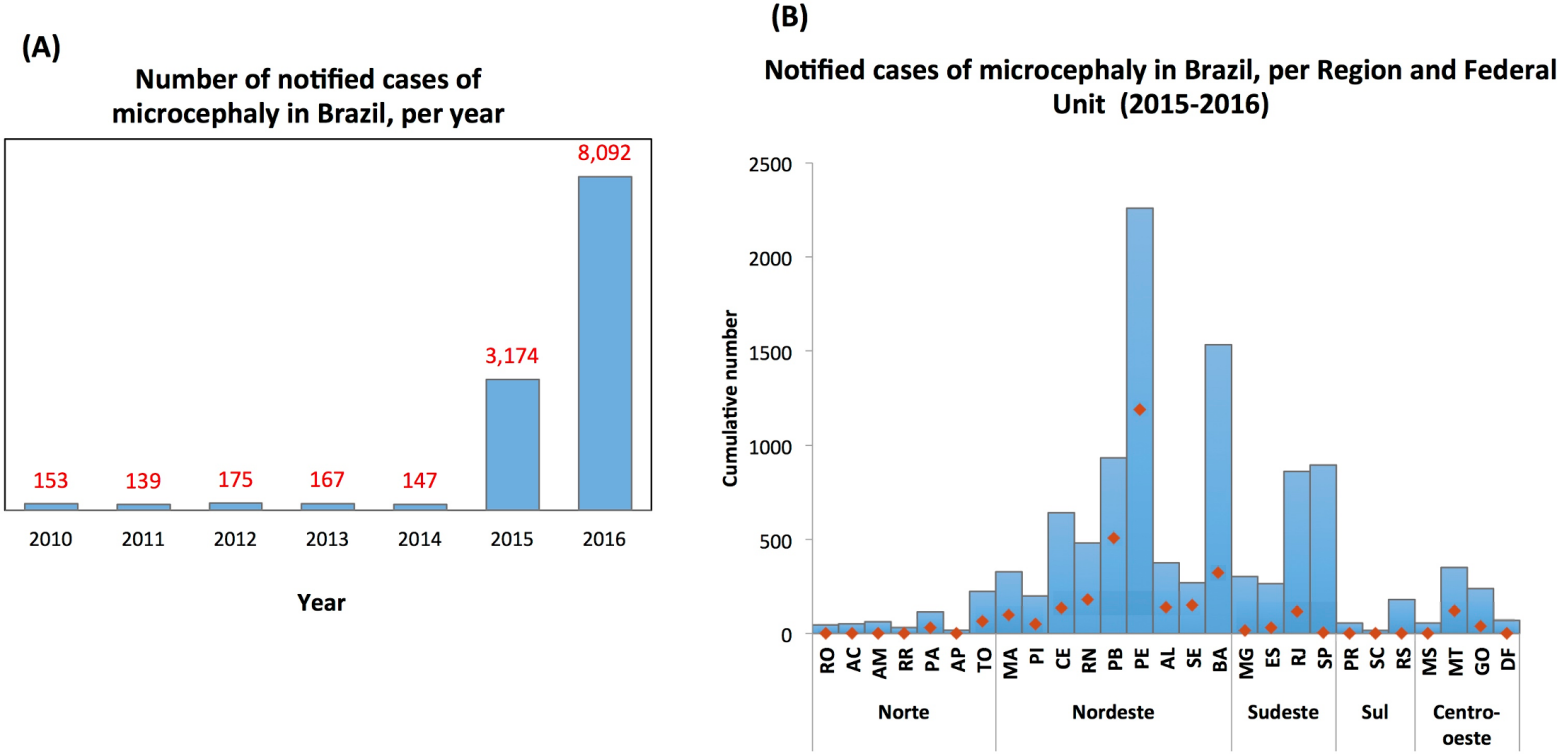
Cases of microcephaly in Brazil, 2010–2016. (A) Total number of notified cases registered in Brazil, per year, from 2010 to 2016; (B) Cumulative number of cases notified in Brazil between 2015 and 2016, per region and Federal Unit. The red line presents values for 2015 only. Notifications were performed based on specific definitions of cephalic perimeter as described in Materials and Methods.

**Fig 2:**
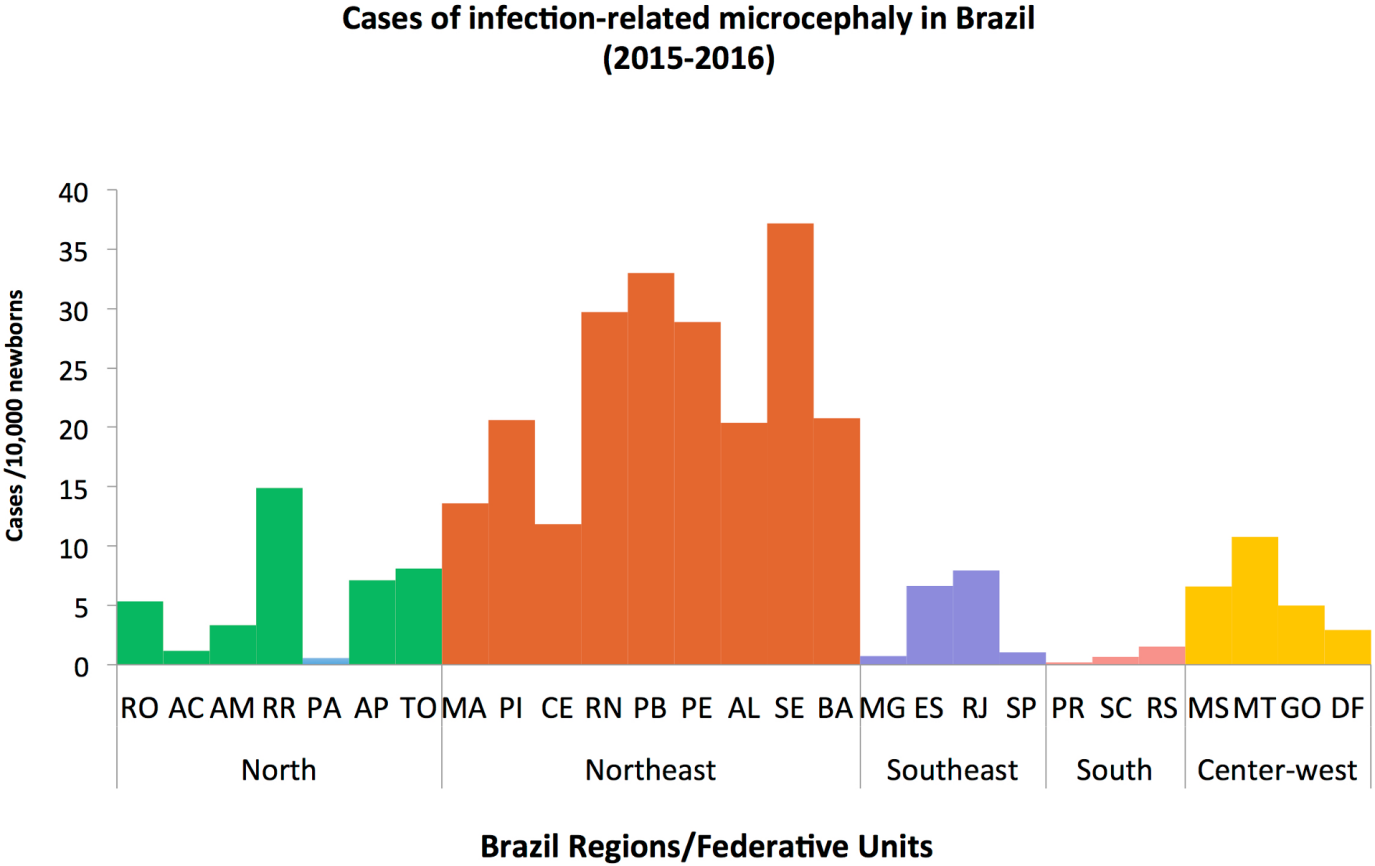
Distribution of infection-related microcephaly cases in Brazil, during the period 2015–2016. Cumulative number of confirmed cases of infection-related microcephaly per 10,000 newborns in Brazil, including clinical or laboratory-confirmed Zika virus infections, during the period 2015–2016. The total number of confirmed infection-related microcephaly cases was normalized by the number of live births extracted from the Information System on Live Births. As there was no live birth data available for 2016, we considered the average number of live births for the last 3 years, for each Brazil Federal Unit.

During the period from 2010–2014, Brazil registered an average of 156 cases of microcephaly per year. Surprisingly, by the end of 2015, the number of cases was 20 times higher (Fig 1A). Pernambuco was the first state reporting the unusual number of microcephaly cases in newborns, with 10 times more notifications than the average for the whole country during the preceding 5 years (Fig 1B) [37]. The number of reported cases kept increasing, with 10,867 notifications reported from November 2015 until the end of 2016, from which 7,023 were registered only in the Northeast region (Fig 1B). Ongoing laboratory investigations confirmed 2,366 positive cases for congenital microcephaly suspected of Zika virus infection or other infectious agents. Of these positive cases, molecular or serological laboratory investigations confirmed Zika infection in 697 microcephaly cases. The top 10 Brazil Federal Units with the highest number of infection-related microcephaly cases includes all the 9 states from the Northeast region and one state from the Northern Brazil (Figs 1 and 2, S1 Fig).

**Fig 3:**
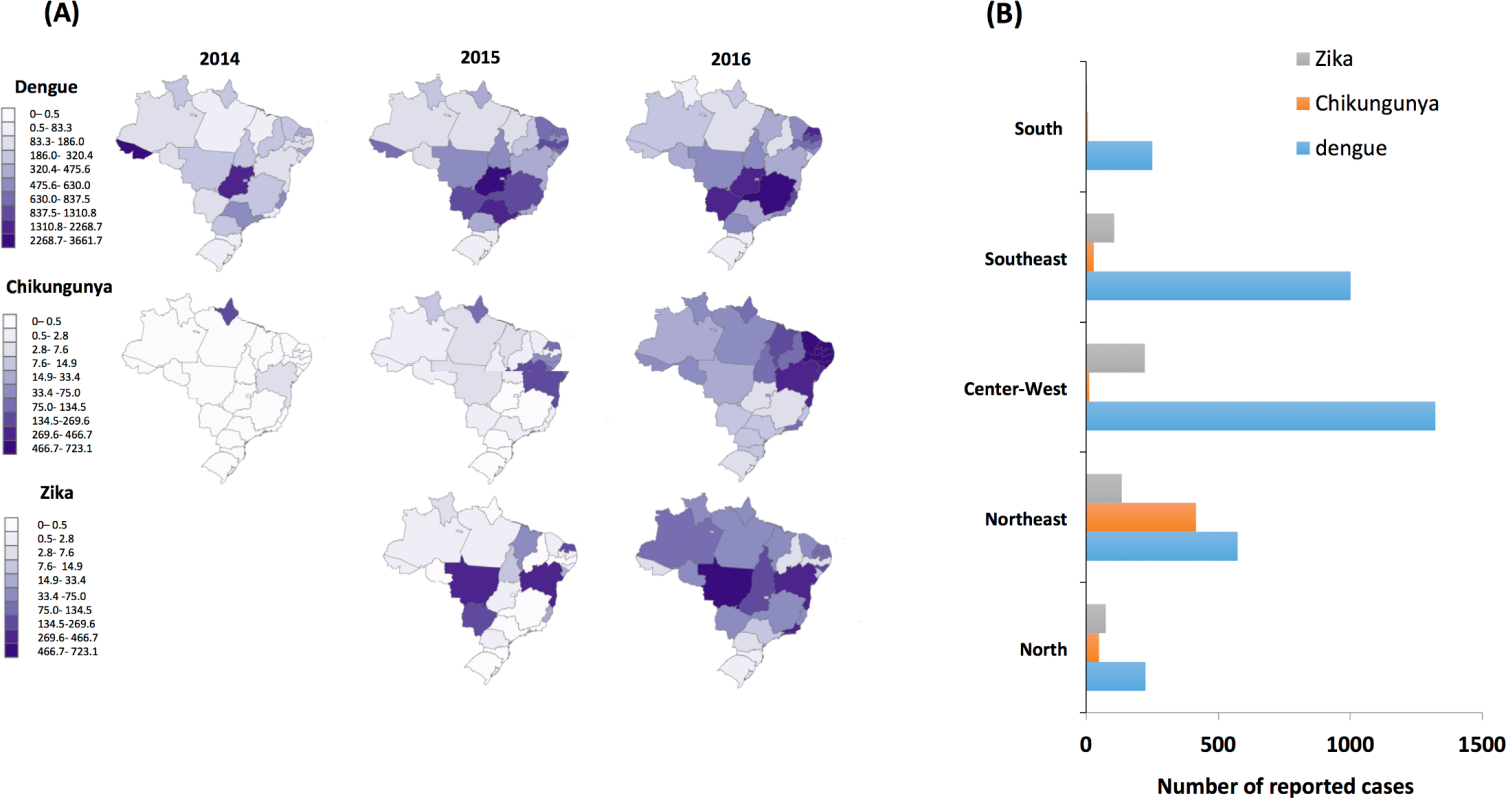
Reported cases of arboviruses in Brazil, per Federal Unit, 2014–2016. (A) Panel shows heat maps for the incidence of dengue, chikungunya and Zika virus per 100,000 inhabitants, in Brazil, from 2014–2016. For the Zika virus, the heat maps start in 2015—the year when the first reports occurred, and includes all suspicious cases, based on epidemiological data collected from Bulletins and reports from the State Secretary of Health of each Federal Unit. (B) Number of reported cases of Zika, dengue and chikungunya in Brazil, per geographical region, during the year 2016.

It was also in the Northeast region that the first cases of Zika infection were reported in May 2015 [4]. However, a study based on the genome of Zika virus strains from Brazil and ecological and epidemiological data, revealed that the Zika virus was already present in the Northeast by February 2014 [38]. By the end of 2015 there was a higher number of suspected Zika cases in the States of Bahia, Mato Grosso, Rio Grande do Norte and Mato Grosso do Sul (Fig 3A). One year later, autochthonous cases of Zika virus infection had been confirmed in all the 27 Brazil Federal Units and more than 200,000 cases have already been registered [5] (Fig 3A). In 2016, the analysis of the reported cases according to geographical regions shows that the Center-West region had the highest incidence rate (222/100,000) followed by Northeast (134.4/100,000) (Fig 3B).

Although it is now established that Zika virus is the cause of severe fetal complications in pregnancy, including microcephaly, the distribution of reported Zika cases and infection-related microcephaly do not overlap (Fig 3A). In fact, there is no correlation between the number of Zika reported cases per 100,000 inhabitants in 2015 or 2016 and the distribution of infection-related microcephaly in Brazil (Parts A and D of S2 Fig) (Correlation R^2^ ≤ 0.25; P ≥ 0.212). During the same period Brazil experienced simultaneous transmission of dengue and chikungunya. During January-September 2016, Brazil recorded 200,465 Zika cases, 236,287 chikungunya cases and 1,438,624 dengue cases [39]. It is possible that co-infection increases the severity of symptoms, as previously shown [40]. In Brazil, few studies have reported the co-circulation and/or co-infection of dengue, Zika, and chikungunya [19, 27, 41]. Interestingly, the number of chikungunya cases reported from 2014 to 2015 (Fig 3A) is predominant in the Northeast region. By 2016 the incidence of chikungunya virus increased in the whole country but it remained mostly concentrated in the Northeast (407.7/100,000) followed by the North (44/100,000) region (Fig 3B). All of the 9 Federal Units from the Northeast region recorded chikungunya cases in excess of 80 per 100,000 habitants. In addition, Amapa and Tocantins states in the North, and Rio de Janeiro in the Southeast, also had a very high number of reported cases (Fig 3A). There is a significant overlap in the distribution of infection-related microcephaly cases and of chikungunya infection (Part B and E of S2 Fig) (R^2^ ≥ 0.57; P ≤ 0.002). There have been reports that associated chikungunya infection with an increase in neurologic manifestations, including Guillain-Barre syndrome and encephalitis, encephalopathy and microcephaly [42–48]. An investigation in the state of Salvador reported the intense chikungunya transmission between June and November 2015 and the co-occurrence with the Guillain-Barre syndrome outbreak in the city [49]. It has also been suggested that the local health authorities have underestimated chikungunya transmission, as attention was focused on the Zika and microcephaly outbreaks [50].

Dengue fever has a wider distribution in Brazil. Between 2014 and 2015, the incidence of dengue increased in almost all geographic regions, keeping similar rates during 2016 (Fig 3A). In the last three consecutive years, the highest number of reported dengue cases has been observed in the Center-West and Southeast regions (Fig 3B). There is no correlation between infection-related microcephaly cases (2015–2016) and the distribution of suspected dengue fever cases (2014–2016) (R^2^ ≤ 0.22, P ≥ 0.272) (Part C and F of S2 Fig). This is consistent with other studies of the dengue virus infection in pregnancy, where although there is evidence of vertical transmission and increased risk of preterm birth and low birthweight [51], no microcephaly or other congenital brain abnormalities have been reported [52].

### Infection-related microcephaly, socio-economic and environmental management factors

**Fig 4:**
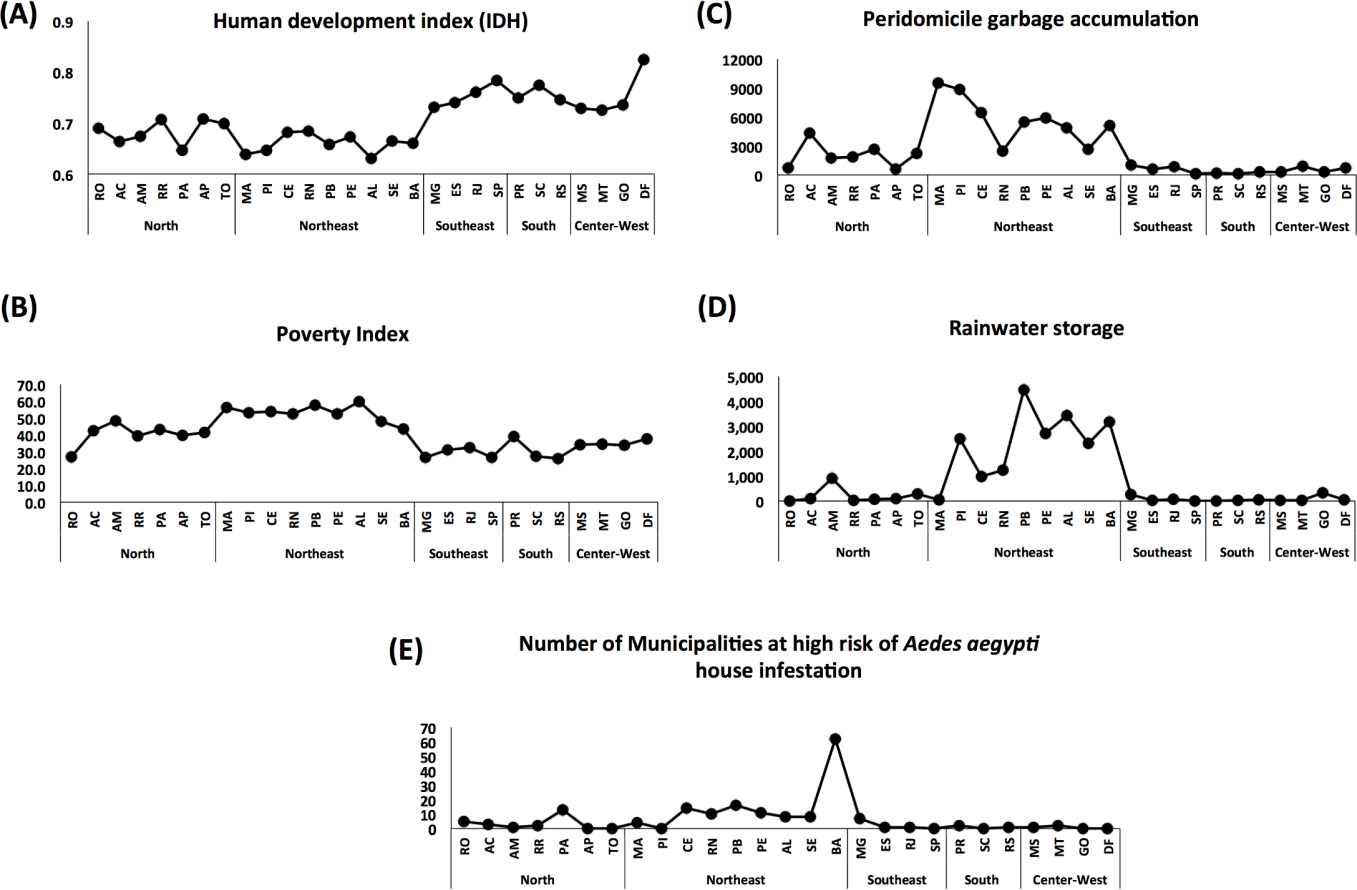
Socioeconomic factors, environmental management and entomological data, per Brazil region. (A) IDH: Human development index (from 0 to 1). Considers education (average years of studies), longevity (population life expectancy) and gross domestic product per capita. (B) Poverty index (from 0 to 100): measures health (nutrition and child mortality), education (school attendance) and living standard (sanitation, water, electricity, cooking fuel, assets, house conditions). (C) Peri-domicile garbage accumulation: number of houses with accumulated and not collected garbage (values per 100,000 houses). (D) Rainwater storage: number of houses that storage rainwater using all different sort of containers (covered or not). (E) Number of Municipalities at high risk of *Aedes aegypti* house infestation: based on the LIRAa survey for the year 2016, which measures the percentage of searched houses found with larvae of *A*. *aegypti*. The figure illustrates the number of municipalities classified as high risk of infestation for that year. A, B, C and D: data from 2010, last available IBGE survey in Brazil.

To evaluate the potential effect of socioeconomic factors on the incidence of Zika-suspected microcephaly, as suggested by others [21–23, 53], we considered the *Human development index* (HDI) and the *poverty index* calculated in 2010. The HDI values range from 0.6 to 0.8 across the country. The Northeast and North regions concentrate the Federal Units with the lowest HDI (= 0.6) (Fig 4A). The highest poverty index is observed in the Northeast region (43.5 to 59.5) (Fig 4B), the epicentre of microcephaly notified cases. There is a strong correlation between the distribution of infection-related microcephaly cases and poverty index (Part A of S3 Fig) (R^2^ = 0.68; p<0.0001).

The Northeast region also had the highest numbers of houses that accumulate garbage and store rainwater (Fig 4C and 4D) that could lead to an increase in vector proliferation and therefore Zika transmission. These results are consistent with a recently reported association of an increase in Zika and chikungunya infections with garbage destination, type of sanitary installation and pipe-borne water [54]. These environmental management indicators may assist an understanding of the population’s behaviours, which may be responsible for increasing the chances of *Aedes* proliferation through underlying human-driven increased mosquito breeding. It also indicates the lack of sanitation and water distribution.

### *Aedes aegypti* house infestation index

In the Americas, the domestic behavior of *Ae*. *aegypti* makes it the most important vector for chikungunya, Zika and dengue virus in urban and suburban areas [55, 56]. A surveillance program of *Ae*. *aegypti* density (house infestation index) was introduced in Brazil aiming to assist the implementation of measures for vector population control. The 2016 *Ae*. *aegypti* house infestation index classified most of the regions in the Northeast region (Ceara, Rio Grande do Norte, Paraiba, Pernambuco, Alagoas, Sergipe, Bahia) or in the North (Para) (Fig 4E), as being at high risk of *Ae*. *aegypti* infestation. In fact, data on the entomological index corroborate the findings on the environmental management in the Northeast region (Fig 4C and 4D). We did observe a correlation across the federal Units of the risk of *Ae*. *aegypti* infestation and the incidence of chikungunya, but not dengue or Zika (S4 Fig) (dengue: R^2^ = 0.01, P = 0.962; chikungunya: R^2^ = 0.44, P = 0.020; zika: R^2^ = 0.25, P = 0.202). It is important to consider that arboviral epidemics are most likely determined by multiple factors including environmental conditions, host status and herd immunity and virus genetic mutations that might cause changes in virulence, or vector competence [57].

## Discussion

Several studies have confirmed the association of Zika infection during pregnancy and congenital microcephaly [58]. Since February 2017, 31 countries or territories have reported microcephaly cases potentially associated with Zika infection [59] However, the overwhelming level of cases in the Brazilian Northeast region has not been reported elsewhere.

During the Zika virus outbreak in Brazil there was simultaneous transmission of dengue and chikungunya. These viral infections are transmitted by the same Aedes spp. mosquitoes and have close clinical manifestations. In Brazil, the prediction values for the distribution and prevalence *of Ae*. *Aegypti*, found mainly in urban environment [55], are higher than for *Ae*. *albopictus*, a predominantly peri-urban and rural vector [60–63]. A study of the geographic distribution of spatially unique occurrence data of Aedes mosquitoes for the Americas shows that *A*. *aegypti* abundance is 1.5 times higher than for *Ae*. *albopictus* in Brazil, and with particularly high concentrations in Northern Brazil [62]. A survey performed in 2014 revealed that *Ae*. *Albopictus* is currently present in at least 59% of the Brazilian municipalities, particularly in the Southeast region [63].

Recent data have shown that in Brazilian urban areas there is an increase number of Zika and chikungunya cases [54], which coincides with the *Ae*. *aegypti* strong affinity to urbanised regions [64]. Conversely, relatively lower numbers of cases occur in forest or agricultural areas [54]. Densely populated areas with poor water infrastructure may provide more breeding opportunities for *Ae*. *Aegypti* [65]. In the Northeast of Brazil, over 75% of the breeding sites are due to precarious water storage [54]. Dengue infections have also been shown to be higher in urban areas in Thailand [66].

Although dengue, chikungunya and Zika viruses share the same vector, there was not a perfect overlap of their spatial and temporal distributions in Brazil. Several factors could lead to the observed distribution, such as herd immunity, vector competence for the different viruses, the proportion of asymptomatic cases and under notification of cases. The Center-West region has the highest reported cases of dengue and Zika virus infections, whereas chikungunya reported infection is much higher in the Northeast region compared to the rest of the country. Chikungunya transmission efficiency seems to be lineage specific. Studies on *Ae*. *aegypti* and *Ae*. *albopictus* from Florida have shown differences in vector competence depending on the chikungunya strain. *Ae*. *albopictus* were more susceptible to infection with the La Réunion strain than sympatric *Ae*. *Aegypti* [67]. A more recent study showed that the chikungunya Asian strain is better transmitted by *Ae*. *aegypti* species than by *Ae*. *albopictus*, whereas the Indian Ocean strain leads to higher body infection and transmission in *Ae*. *albopictus* mosquitoes [68]. In addition, virus mutations can interfere with the transmission rate, for instance, an alanine-valine substitution at position 226 of the E1 envelope glycoprotein (E1-A226V) can improve transmission by *Ae*. *albopictus* [69, 70] Comparative genome studies on the chikungunya virus circulating in different Brazil regions could provide insights on the higher number of chikungunya cases found in the Northeastern Brazil.

The initial epidemic of the Zika virus was also reported in the Northeast region, spreading eventually to other states. Few studies in Brazil have reported the co-circulation and/or co-infection of dengue, Zika, and chikungunya in the Northeast region [19, 41]. In Bahia, a small study using metagenomic next-generation sequencing approach revealed that 13.3% of patients with confirmed Zika virus infection were actually co-infected with chikungunya virus [27]. Other studies worldwide have also reported co-infections with various combinations of chikungunya/dengue and Zika in endemic and epidemic regions [71–72]. It has also been shown that *A*. *aegypti* mosquitoes can co-transmit all combinations of these viruses simultaneously without affecting vector competence [73]. The effect of co-infections in the development of infection and disease outcome is poorly defined, particularly due to the limited clinical information, misdiagnosis and lack of laboratory testing. However, some studies have indicated that previous arboviral infections or co-infections may represent a risk factor for severe clinical manifestations [26]. Pre-existing anti-dengue immunity can promote substantial enhancement of Zika virus infection *in vitro* as well as increased morbidity and mortality in mice [40]. Also, dengue-specific antibodies enhance the infection of a primary Brazilian Zika isolate in K562 cell line [74]. Even though our data did not show an association of infection-related microcephaly cases and the distribution of dengue fever, it is important to note that dengue is endemic in the Northeast region, with 573.3 reported cases per 100,000 inhabitants in 2016 [39]. Dengue transmission is permanent in all regions where Zika and chikungunya infection were reported [75], and there is a high probability of previous or co-infection of dengue with either virus. In relation to the observed outbreaks of chikungunya and Zika, these infections partially overlap by time and space [76], as described in more detail for regions in the Northeast including Pernambuco, Recife [77, 78] and Bahia [49], indicating that previous or co-infection of these virus is possible. It is important to note that the overlap in space-time distribution of Zika, dengue, and chikungunya cases is challenging to report, particularly due to the number of asymptomatic cases and the possible presence of non-specific clinical manifestations which are difficult to diagnose.

The pathogenic effect of Zika and chikungunya virus co-infection, or of the three viruses, has not been studied. We observed a correlation between the distribution of chikungunya infection and the congenital microcephaly in the Northeast region, which may indicate that previous arboviral infections or co-infection with chikungunya could increase Zika severity. Others have linked the outbreak of Guillain-Barre syndrome, firstly related to the Zika virus outbreak in 2015 in the city of Salvador, with the concurrent intense chikungunya transmission [50]. It is important to note that chikungunya infection can result in neurologic manifestations such as encephalitis [79], encephalopathy [43,80], peripheral neuropathy (including Guillain-Barre syndrome) [46,81–82]. In addition, chikungunya virus infection may result in complications during pregnancy either to the mother or to the newborn [83–84]. Complications for the mother, such as chronic inflammatory rheumatism, as well as neurocognitive impairment in infants and microcephaly have been reported in congenital chikungunya transmission [47,49,85–86].

The Northeast region of Brazil has the highest poverty index, with the lowest social economic factors and poorest environmental management, which can be responsible for increasing *Aedes* proliferation. Poverty can drive malnutrition and general poor health that might affect host immunity and the response and clinical progression of an infection.

Altogether, these findings corroborate previous study that suggests that the Zika virus may not be the only factor responsible for the high frequency of congenital microcephaly observed in the Northeast region [55]. Here, using ecological data analysis, we highlight the possible co-circulation of the three arboviruses, together with the socio-economic and environmental factors specific for that region. To understand the complete pathogenesis and severity of Zika infection in the presence of other viruses and other possible co-factors, it is necessary to conduct comprehensive cohort studies involving large patient groups, with detailed socio-economic and environmental factors and effective differential diagnosis for these viruses.

### Limitations of the study

It is important to consider that the findings in this study are subject to at least six limitations. Firstly, the cases of infection-related microcephaly reported in the databases include congenital microcephaly caused by Zika virus or other infectious agents. Therefore, it is not known if the distribution of microcephaly cases is caused by laboratory confirmed Zika virus infection. Secondly, cases of co-infections (dengue, Zika, chikungunya) were not investigated or not reported. Third, the ascertainment of birth defects generally does not capture infants or fetuses whose birth defects are not apparent prenatally or at delivery, but rather are identified several months after birth. Fourth, there was no report on *Ae*. *albopictus* distribution by the LIRAa survey. Fifth, the molecular characterization and phylogenetic analysis of the current circulating strains of Zika, dengue and chikungunya viruses in Brazil is important, but was not possible. Sixth, these data are analyzed at a State-level, which are large regions, and differences between smaller areas (municipalities) were not investigated. Future studies of the genomic epidemiology of these viruses can assist with improving an understanding of the biology, disease phenotypes and transmission, and support the design of diagnostic and vaccine strategies to control the next epidemics.

Overall, we analysed a set of robustly collected and curated data on epidemiological, environmental and socio economic factors of all regions in Brazil. Our results support the hypothesis that the high rates of microcephaly in the Northeast state in Brazil might not be due to Zika infection alone. Our study reinforces the need for comprehensive large-scale cohort studies, as well as public-health measures and guidance to better inform the population under the higher risk of infection-related microcephaly in Northeast of Brazil.

### Conclusions

The unusual distribution of microcephaly-Zika associated cases in Brazil is likely to be caused by a combination of epidemiological, environmental and socio economic factors. Our work highlights the overlap between the distribution of chikungunya infection with the co-incidence of infection-related microcephaly in the Northeast region. It emphasizes the link between poverty and the risk of disease, and the impact that poor environments can have on human health and the spread of infections. To understand the impact of co-infection on disease outcomes, we recommend that the list of infectious diseases screened during epidemics, particularly for pregnant woman, is updated to include and effective differentiate arboviruses from ongoing outbreaks or epidemics.

## Supporting information

S1 Fig A map of Brazil highlighting regional divisions and Federal Units. (JPG)

S2 Fig Association between infection-related microcephaly and incidence of arboviruses. Pearson’s correlation test and linear regression was used to investigate the association between infection-related microcephaly and incidence of: Zika in 2015 (A) and 2016 (D); chikungunya in 2015 (B) and 2016 (E); dengue in 2015 (C) and 2016 (F); all per region of Brazil, and results were considered significant for P<0.05.
(JPG)

S3 Fig The correlation between infection-related microcephaly and poverty index. Microcephaly versus poverty index. Results were considered significant when P<0.05.
(JPG)

S4 Fig The correlation between *Aedes aegypti* infestation risk (LIRAa) and arboviruses incidence in the year 2016. The number of municipalities at high risk of *A*. *aegypti* infestation, per Brazil Federal Unit, versus incidence of dengue (A), chikungunya (B) and Zika (C). Linear correlation was considered significant when P<0.05.
(JPG)
