# Supplementary figures and images for: Zika might not be acting alone: Using an ecological study approach to investigate potential co-acting risk factors for an unusual pattern of microcephaly in Brazil
Source: PLoS One. 2018 Aug 15;13(8):e0201452. doi: 10.1371/journal.pone.0201452 (PMC6093667; doi:10.1371/journal.pone.0201452)

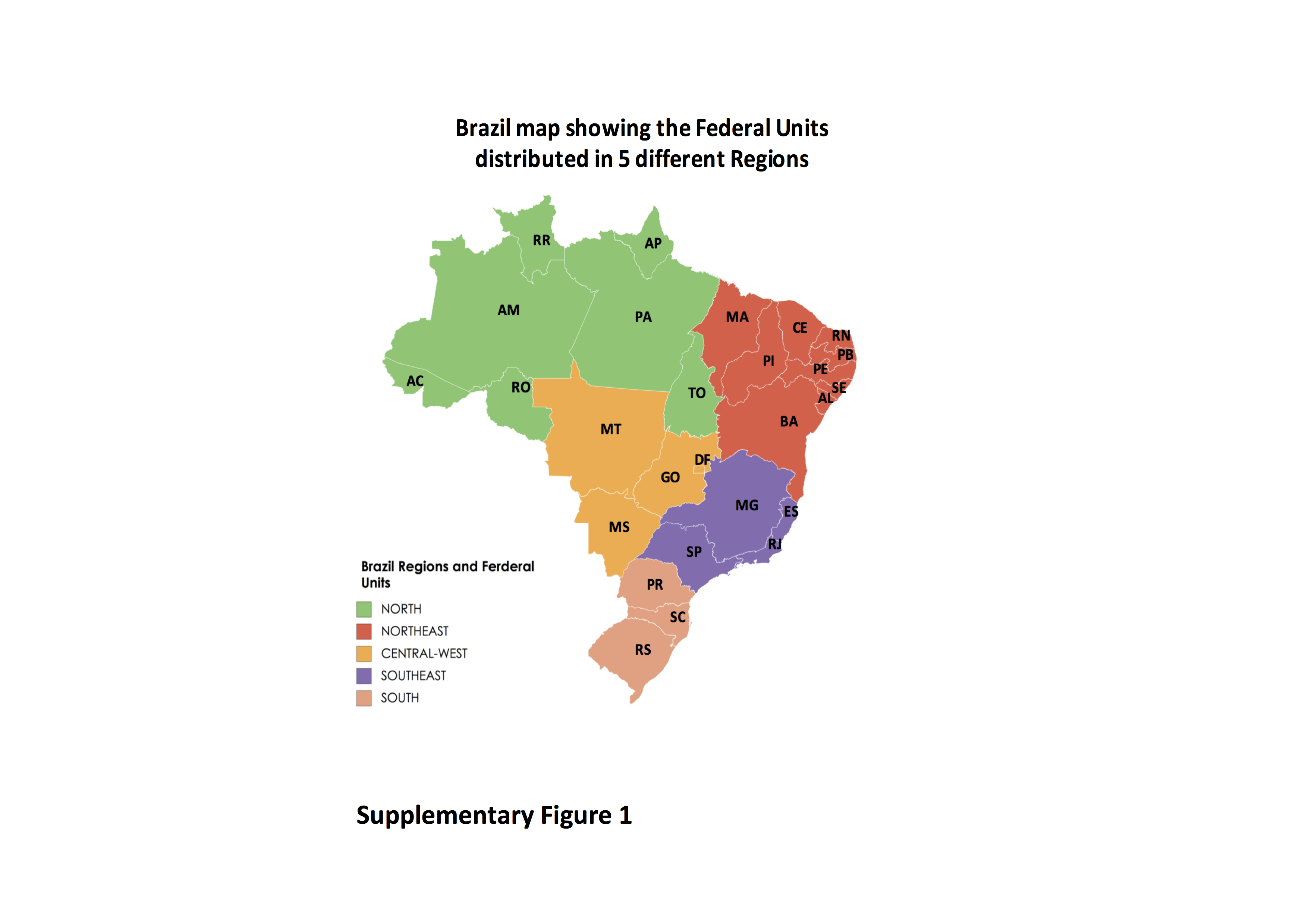

Supplement: S1 Fig — (JPG) [file pone.0201452.s001.jpg]

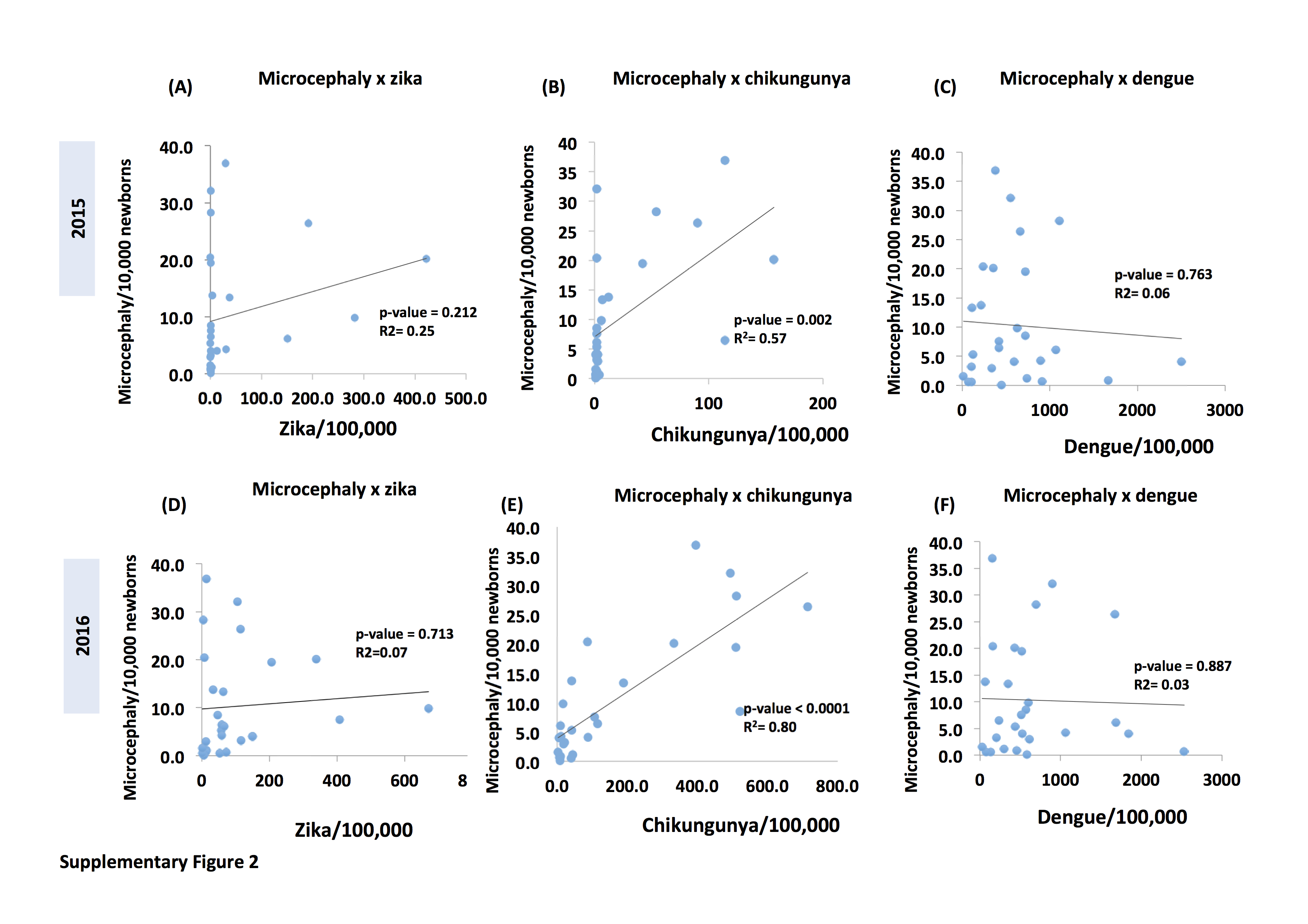

Supplement: S2 Fig — Pearson’s correlation test and linear regression was used to investigate the association between infection-related microcephaly and incidence of: Zika in 2015 (A) and 2016 (D); chikungunya in 2015 (B) and 2016 (E); dengue in 2015 (C) and 2016 (F); all per region of Brazil, and results were considered significant for P<0.05. (JPG) [file pone.0201452.s002.jpg]

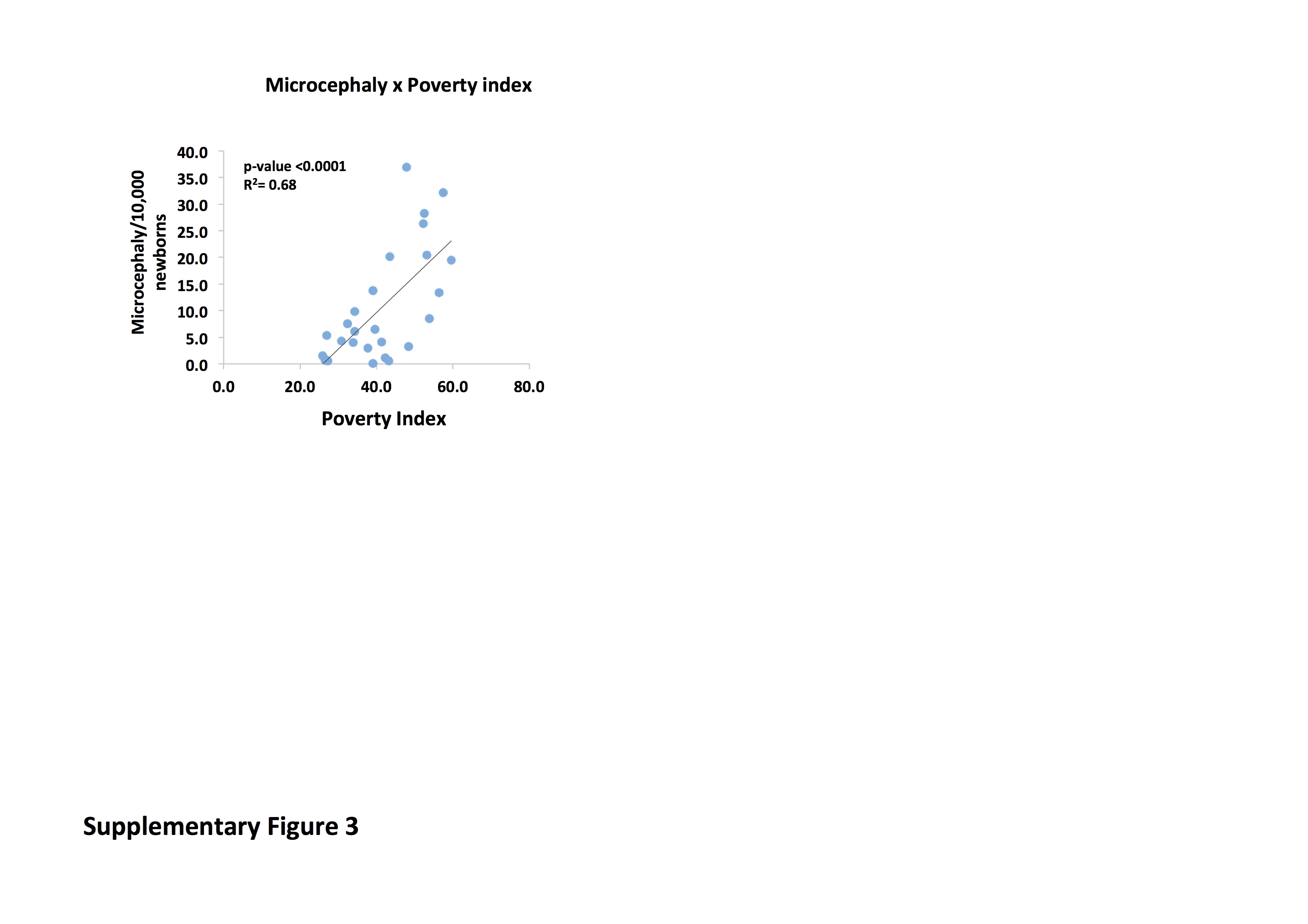

Supplement: S3 Fig — Microcephaly versus poverty index. Results were considered significant when P<0.05. (JPG) [file pone.0201452.s003.jpg]

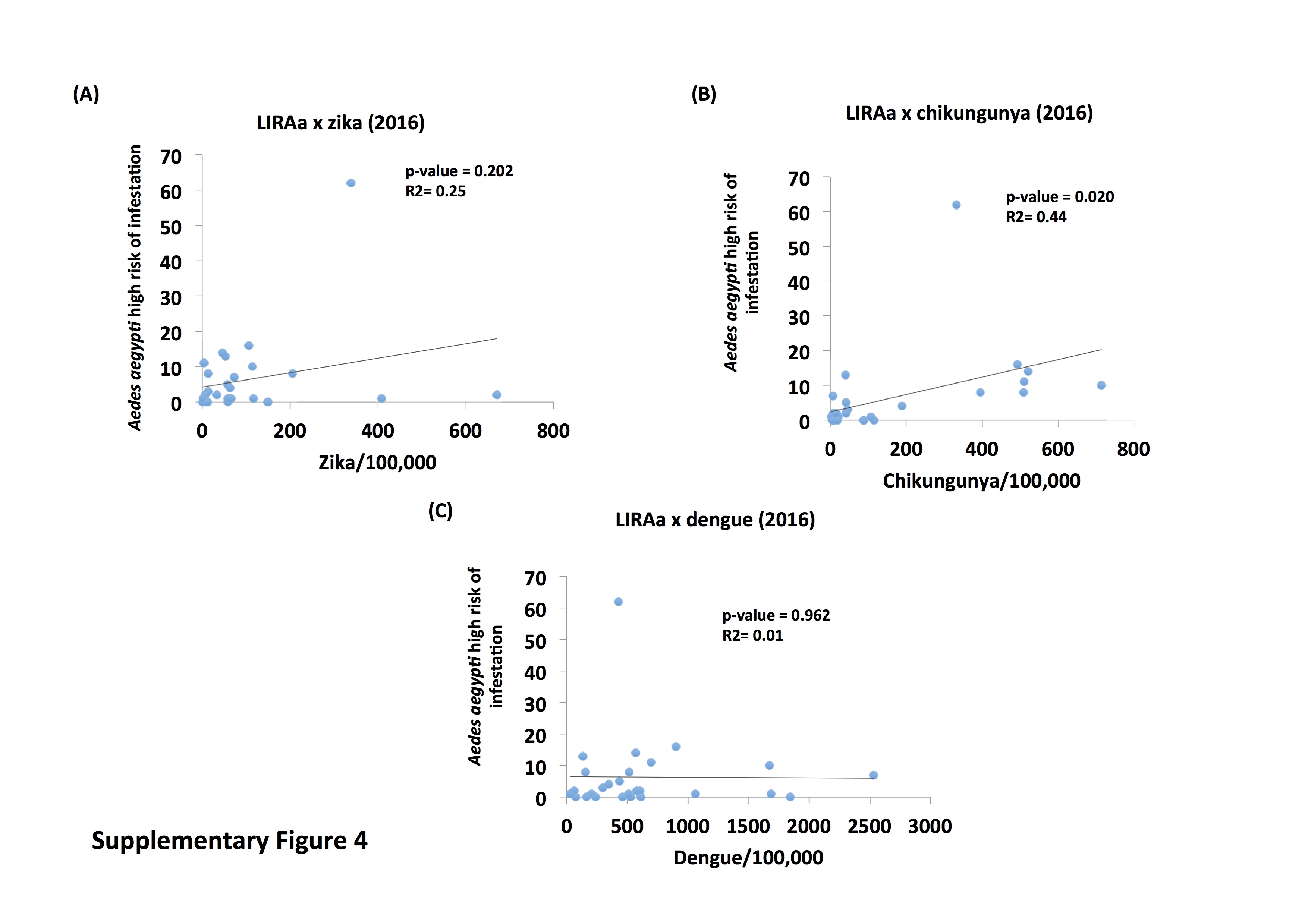

Supplement: S4 Fig — The number of municipalities at high risk of A. aegypti infestation, per Brazil Federal Unit, versus incidence of dengue (A), chikungunya (B) and Zika (C). Linear correlation was considered significant when P<0.05. (JPG) [file pone.0201452.s004.jpg]
